# Whole-body Computed Tomography Versus Dual Energy X‑ray Absorptiometry for Assessing Heterotopic Ossification in Fibrodysplasia Ossificans Progressiva

**DOI:** 10.1007/s00223-021-00877-6

**Published:** 2021-07-31

**Authors:** Sarah E. Warner, Frederick S. Kaplan, Robert J. Pignolo, Stacy E. Smith, Edward C. Hsiao, Carmen De Cunto, Maja Di Rocco, Kathleen Harnett, Donna Grogan, Harry K. Genant

**Affiliations:** 1 Scientific and Medical Services, PAREXEL International (dba Calyx), Billerica, MA USA; 2 Departments of Orthopaedic Surgery & Medicine, The Center for Research in FOP and Related Disorders, Perelman School of Medicine, University of Pennsylvania, Philadelphia, PA USA; 3 Department of Medicine, Mayo Clinic, Rochester, MN USA; 4 Division of Musculoskeletal Imaging and Intervention, Department of Radiology, Brigham and Women’s Hospital, and The Neil and Elise Wallace STRATUS Center for Medical Simulation, Harvard Medical School, Boston, MA USA; 5 Division of Endocrinology and Metabolism, the UCSF Metabolic Bone Clinic, and the Institute of Human Genetics, Department of Medicine, and the UCSF Program in Craniofacial Biology, University of California-San Francisco, San Francisco, CA USA; 6 Pediatric Rheumatology Section, Department of Pediatrics, Hospital Italiano de Buenos Aires, Buenos Aires, Argentina; 7 Unit of Rare Diseases, Department of Pediatrics, Giannina Gaslini Institute, Genoa, Italy; 8 Ipsen, Newton, MA USA; 9 Departments of Radiology, Medicine and Orthopaedic Surgery, University of California, San Francisco, CA USA

**Keywords:** Fibrodysplasia ossificans progressiva, Whole-body computed tomography, Dual energy X-ray absorptiometry, Heterotopic ossification

## Abstract

Fibrodysplasia ossificans progressiva (FOP) is an ultra-rare genetic disorder that leads to heterotopic ossification (HO), resulting in progressive restriction of physical function. In this study, low-dose, whole-body computed tomography (WBCT) and dual energy X-ray absorptiometry (DXA) were evaluated to determine the preferred method for assessing total body burden of HO in patients with FOP. This was a non-interventional, two-part natural history study in patients with FOP (NCT02322255; date of registration: December 2014). In Part A (described here), WBCT and DXA scans were individually assessed for HO presence and severity across 15 anatomical regions. All images were independently reviewed by an expert imaging panel. Ten adult patients were enrolled across four sites. The sensitivity to HO presence and severity varied considerably between the two imaging modalities, with WBCT demonstrating HO in more body regions than DXA (76/138 [55%] versus 47/113 [42%]) evaluable regions). Inability to evaluate HO presence, due to overlapping body regions (positional ambiguity), occurred less frequently by WBCT than by DXA (mean number of non-evaluable regions per scan 1.2 [standard deviation: 1.5] versus 2.4 [1.4]). Based on the increased sensitivity and decreased positional ambiguity of low-dose WBCT versus DXA in measuring HO in patients with FOP, low-dose WBCT was chosen as the preferred imaging for measuring HO. Therefore, low-dose WBCT was carried forward to Part B of the natural history study, which evaluated disease progression over 36 months in a larger population of patients with FOP.

## Introduction

Fibrodysplasia ossificans progressiva (FOP; OMIM #135,100) is an ultra-rare genetic disorder with an estimated global prevalence of 1.36 per million individuals [1, 2]. Approximately 97% of patients with FOP have an R206H mutation in the gene activin A receptor type I (*ACVR1;* also known as activin-like kinase 2 [*ALK2*]) [3–5]. The condition is characterized by congenital skeletal malformations and extra-skeletal bone formation in muscles, tendons, ligaments, and aponeuroses, referred to as heterotopic ossification (HO). HO leads to progressive joint ankylosis, which restricts movement and physical function and leads to significant deterioration in quality of life [6]. Most patients are confined to a wheelchair by the third decade of life and require lifelong assistance with routine activities [7].

There are currently no effective treatments to prevent the formation of heterotopic bone in FOP, and medical intervention is limited to supportive care and management of flare-ups [8–11]. Non-steroidal anti-inflammatory agents (NSAIDs) and short-term use of high-dose corticosteroids may be used for symptomatic alleviation [12].

To support the development of therapies for FOP, it is critical to be able to elucidate the natural history of the disease, particularly in terms of HO progression throughout the body. Therefore, a two-part natural history study (NHS) was designed, which aimed to describe disease progression over 36 months in patients with FOP (NCT02322255) [13]. A key challenge to the design of the NHS was determining the optimal imaging modality for assessing the progression of HO over time, which was investigated in Part A of the NHS. Feasibility factors included cost, availability, benefit, invasiveness, burden, practicality, ability to standardize across multiple centers, and ability to accommodate patients with significantly ankylosed joints and limited mobility. The preferred modality was used to assess the progression of FOP in Part B of the NHS.

Various imaging modalities can be used to evaluate HO burden in patients with FOP [14]. Previous studies have reported the use of site-specific radiographs, radionuclide bone scans, magnetic resonance imaging (MRI) and computed tomography (CT) scans [15–18]. Positron emission tomography (PET)-CT has recently also been used to image HO in FOP [19]. Very few studies have reported using imaging to assess progression of HO in patients with FOP over time [20], which would provide clinicians and patients with a better understanding of the natural progression of disease, and specifically the extent of and, ideally, quantitative changes in HO.

At the time the NHS was designed, most prior imaging in FOP was limited to site-specific, rather than whole-body imaging. Several whole-body imaging modalities have been used to evaluate total body burden of HO over time, including radiographic skeletal surveys, whole-body X-ray (slit-beam digital radiography system), radionuclide bone scans, dual energy X-ray absorptiometry (DXA), whole-body CT (WBCT), and CT scout scans. Many of these imaging modalities have limitations including radiation exposure (radiographic skeletal surveys), limited availability (slit-beam digital radiography system), low resolution (bone scans) and practical constraints to carrying out scans (radiographic skeletal surveys, slit-beam digital radiography system, MRI).

After taking these factors into consideration, two whole-body imaging modalities (low-dose WBCT and DXA) were selected for further evaluation in Part A of the NHS. Our objective was to determine the preferred method for assessing total body burden of HO, considering sensitivity in characterizing HO, radiation exposure, and practicality of imaging patients with FOP-related deformities. Results of the evaluation are presented here.

## Methods

### Patients and Study Design

The NHS in FOP was a multicenter, non-interventional, longitudinal, two-part study (NCT02322255) in patients with classic FOP (*ACVR1*^R206H^) carried out between December 18 2014 and April 9 2020. Patients in Part A were enrolled across four sites globally (University of Pennsylvania, Philadelphia, PA, USA; University of California San Francisco, San Francisco, CA, USA; Gaslini Institute, Genoa, Italy; and Hospital Italiano de Buenos Aires, Buenos Aires, Argentina), following a thorough baseline examination to determine their current disease state. Individuals unable or unwilling to complete study-related procedures (including radiographic assessments) were ineligible.

In Part A of the NHS, enrollment was restricted to patients ≥ 18 years of age. At baseline, patients underwent both low-dose WBCT and DXA scans to determine the preferred imaging modality (Table 1). The selected modality was then carried forward into Part B to evaluate FOP disease progression over 36 months in a larger population of patients (aged ≤ 65 years). Here, data are reported from Part A of the NHS.

**Table 1:** Characteristics of WBCT and DXA

	Low-dose WBCT	DXA
Image dimensionality	3D	2D
Presence and change in HO	Qualitative and quantitative (volume)	Qualitative (area measurement not possible due to insufficient sensitivity to differentiate normotopic bone from heterotopic bone)
Anatomic data	Presence of e.g., dysplasias, renal stones can be detected	Anatomic data not easily detected
HO severity assessment criteria	Mild/moderate/severe	Mild/moderate/severe
Quantitative total body burden of HO	Yes (total HO volume)	No
Assessment of number of body regions with HO	Yes	Yes
Patient burden^a^	Minimal	Minimal

*DXA* dual energy X-ray absorptiometry, *mSv* millisievert, *WBCT* whole-body computed tomography

^a^Based on a qualitative assessment

### Image Acquisition

To harmonize the acquisition of low-dose WBCT and DXA scans across sites, guidelines and training were provided to all imaging technologists prior to the start of the NHS. A single, independent, American Board of Radiology-certified musculoskeletal radiologist served as the reviewer for all imaging in Part A of the NHS. As Part A of this NHS was the pilot phase, it was deemed sufficient to have a single, independent radiologist review the images during this part of the study. WBCT and DXA images were processed for quality assurance and presented to the reviewer in a blinded fashion (masking of site and patient identifiers) using a standardized electronic case report form and image viewing software (Alice v9.0, Calyx, Billerica, MA).

Due to the nature of the characteristic deformities in FOP, patients were transferred and positioned carefully to optimize imaging, limit discomfort, and prevent trauma that could potentially exacerbate FOP. Sufficient padding was supplied to patients where necessary, and care was taken to ensure adequate clearance of imaging equipment.

#### Whole-body Computed Tomography (WBCT)

Low-dose WBCT (excluding head) scout views were acquired in coronal and sagittal planes. Axial scans were acquired in the cranio-caudal direction from the base of the skull to the feet, using 3 mm axial slices with 512 × 512 matrix and pitch of 1. Bone and soft-tissue kernels were utilized, and coronal and sagittal reconstructions were generated. All sites were advised to utilize As Low As Reasonably Achievable (ALARA) principles and make every effort to reduce the radiation exposure [21]. Radiation exposure reductions were attained using reduced tube voltage, automated tube current modulation for body size/habitus, and iterative reconstruction algorithms.

Following completion of low-dose WBCT, the acquisition parameters for each scan were evaluated to determine the estimated radiation exposure. The kilovoltage peak (kVp), CT Dose Index volume (CTDIv), Dose Length Product (DLP), and patient age and sex were extracted. Available imaging data from the online DICOM database were evaluated by a medical physicist to determine the scan length for the neck, chest, abdomen/pelvis, and lower extremities in each CT scan, and to determine the individual DLP values for each body section.

#### Dual Energy X-ray Absorptiometry (DXA)

DXA scans were acquired on either Hologic (*n* = 1) or GE Lunar scanners (*n* = 3). The sites were instructed to position the patients according to the manufacturer’s recommendations, so that the whole body was within the maximum scan field of view with the patient in supine position. Standard DXA whole-body scan protocols were used. Scans were submitted to the imaging core lab (PAREXEL Informatics dba Calyx, Billerica, MA) for independent review. To enable image review outside of the DXA proprietary analysis software, the scans were converted to DICOM images and presented in a standardized fashion to the independent reviewer for qualitative determination of presence, location and severity of HO across 15 body regions (described below).

### Image Review

**Fig. 1:**
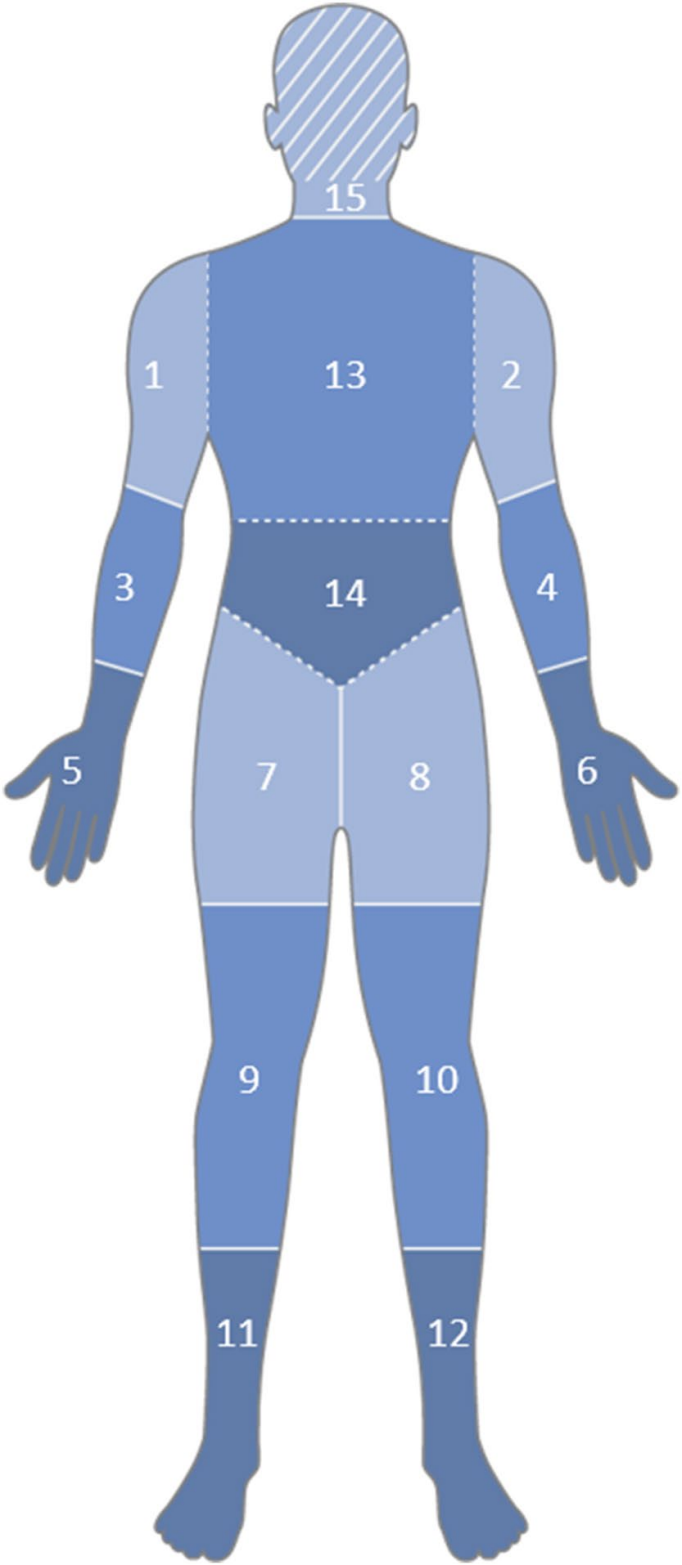
Body regions assessed for the presence of HO. Figure depicts the anatomical regions assessed for HO: (1) the right shoulder (shoulder through mid-humerus); (2) the left shoulder (shoulder through mid-humerus); (3) the right elbow (mid-humerus through mid-radius/ulna; (4) the left elbow (mid-humerus through mid-radius/ulna); (5) right distal upper extremity (mid-radius/ulna including entire hand); (6) left distal upper extremity (mid-radius/ulna including entire hand); (7) right hip (entire hip, including iliac crest and femoral head through mid-femur; (8) left hip (entire hip, including iliac crest and femoral head through mid-femur); (9) right knee (mid-femur through mid-tibia); (10) left knee (mid-femur through mid-tibia); (11) right distal lower extremity (mid-tibia [or distal], including whole foot); (12) left distal lower extremity (mid-tibia [or distal], including whole foot); (13) upper spine/chest (thoracic spine); (14) lower spine/abdomen (lumbar spine); (15) head and neck (for WBCT, the head was not included). *DXA* dual energy X-ray absorptiometry, *HO* heterotopic ossification, *WBCT* whole-body computed tomography

The independent review of each DXA scan was completed before that of the low-dose WBCT scan for each patient. All scans were individually assessed for the presence (yes/no/not evaluable) of HO in 15 body regions: neck, chest, abdomen, and three sub-regions of each leg and arm (proximal, mid and distal) (Fig. 1). Anatomical regions with HO were then scored qualitatively for severity of HO as mild/moderate/severe, depending on the proportion of adjacent soft tissue showing evidence of HO (Mild: very small proportion of the region shows evidence of HO; Moderate: moderate proportion of region includes HO, or longest diameter of contiguous HO in the region appears to be at least half the diameter of the reference normotopic bone in that region; Severe: large proportion of the region includes HO or longest diameter of contiguous HO in the region appears to be equal to or greater than the diameter of the reference normotopic bone in that region). In regions where insufficient image quality or anatomical coverage prevented determination of HO presence, results were reported as ‘not evaluable’.

**Fig. 2:**
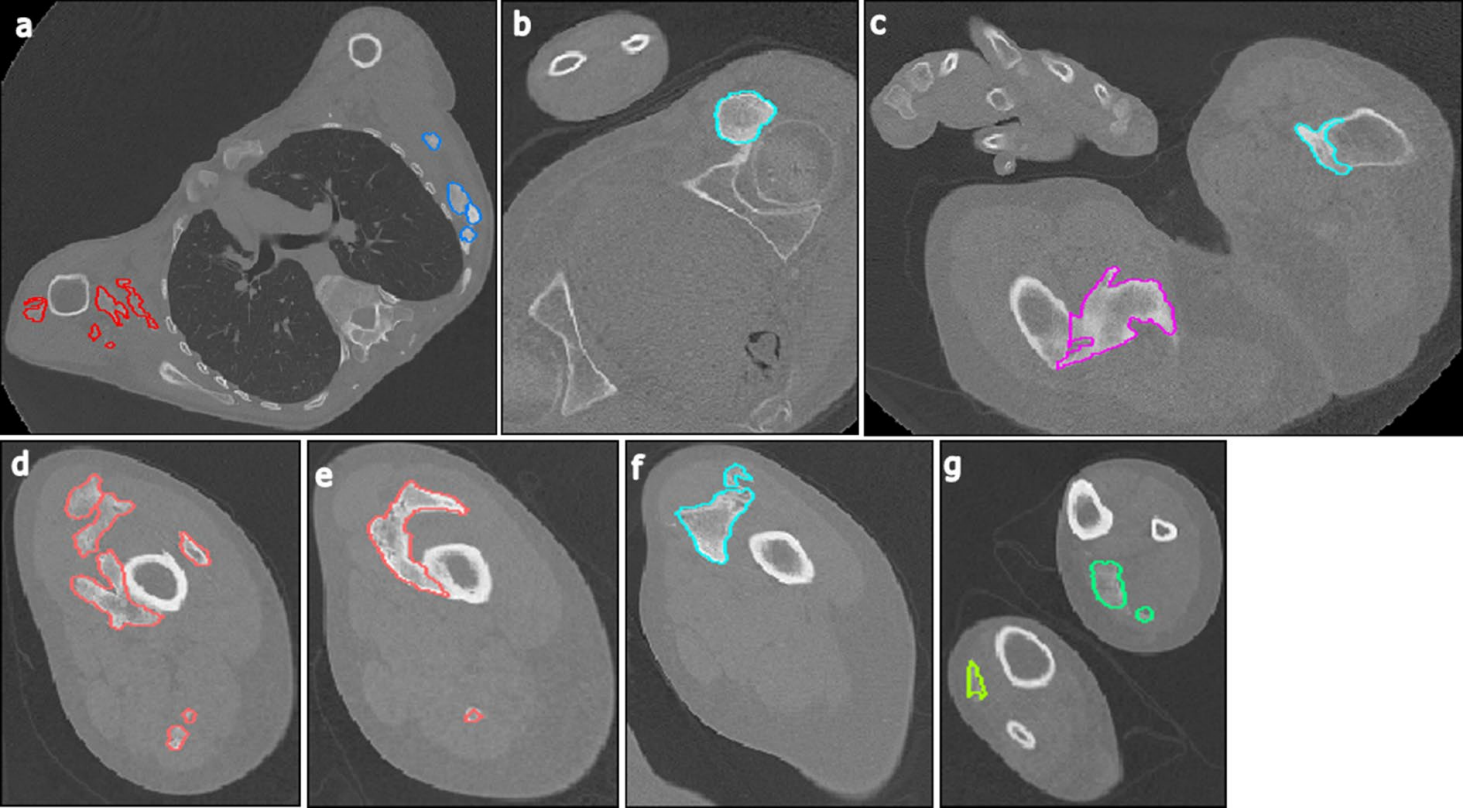
Representative 3-mm axial slices from a CT scan showing HO segmentations. Images were taken from the same 37-year-old male, with a Baseline CAJIS score of 24 at enrollment. Axial slices depict HO (each lesion segmented with a different color) located in: **a** upper body regions (representing regions 1, 2 and 13 from diagram in Fig. 1); **b** anterior to the left hip (region 8); **c** left and right hips (regions 7 and 8); **d**/**e** the right upper leg (region 9); **f** the left upper leg (region 10); and **g** the distal lower legs (regions 11 and 12). *CAJIS* Cumulative Analogue Joint Involvement Scale for FOP, *CT* computed tomography, *HO* heterotopic ossification

To determine total HO volume by low-dose WBCT, HO was segmented on each axial slice (Fig. 2). Segmentations were performed using semi-automated seed growing and shrink wrap segmentation algorithms based on Hounsfield Units whenever possible; otherwise, the radiologist reviewer used manual contouring and nudging steps (Alice v9.0, Calyx, Billerica, MA) to optimize the HO segmentations based upon visual confirmation of calcified tissue voxels. The HO volumes were calculated separately for each of the 15 body regions and summed to provide the whole-body burden of HO volume.

Following independent review of WBCT and DXA scans, the images and data were reviewed by an expert panel to determine the optimal imaging modality for the remainder of the NHS (Part B). This panel was comprised of the original independent reviewer; a second, independent musculoskeletal radiologist; the global Principal Investigator; and the study sponsor representative(s). The committee reviewed the available images, the reviewer’s primary read results, and clinical information for each patient (including demographics, FOP history, and range of motion using the Cumulative Analogue Joint Involvement Scale [CAJIS] [22]) to determine the most appropriate modality for assessing HO burden in Part B of the NHS.

### Data Analysis

Descriptive statistics were used for patient demographics, radiation exposure and HO incidence. No quantitative statistical analyses were performed due to low patient numbers. A qualitative analysis was performed by the authors and the decision to favor low-dose WBCT over DXA was made by expert opinion.

## Results

### Patient Characteristics and Radiation Exposure

Ten adult patients with FOP were enrolled across the four sites in Part A (University of Pennsylvania, Philadelphia, PA, USA: *n* = 3; University of California San Francisco, San Francisco, CA, USA: *n* = 1; Gaslini Institute, Genoa, Italy: *n* = 1; Hospital Italiano de Buenos Aires, Buenos Aires, Argentina: *n* = 5). Demographics and clinical characteristics are described in Table 2. Half the enrolled patients were female, with a mean age of 28.6 years (standard deviation [SD]: 5.7) and a median CAJIS score of 19.0 (range 10–26). After enrollment, DXA images could not be obtained from one patient who was unable to fit in the scanner due to the positioning of a limb.

**Table 2:** Patient demographics and disease characteristics

Characteristic	N = 10
Patients with low-dose WBCT scans, n (%)	10 (100)
Patients with DXA scans, n (%)^a^	9 (90)
Female, n (%)	5 (50)
Age, years, mean (SD); median (range)	28.6 (5.7); 29.0 (18–37)
Weight, kg, mean (SD); median (range)	62.1 (17.7); 61.1 (43–107)
Height, cm, mean (SD); median (range)	162.2 (11.8); 162.5 (144–179)
CAJIS score, mean (SD); median (range)^b^	18.1 (6.2); 19.0 (10–26)
Age at first flare-up, years, mean (SD); median (range)	8.3 (6.2); 5.5 (2–17)
Flare-ups in the past 12 months, mean (SD); median (range)^c^	2.1 (2.0); 2.0 (1–4)

*CAJIS* Cumulative Analogue Joint Involvement Scale, *DXA* dual energy X-ray absorptiometry,* FOP* fibrodysplasia ossificans progressive, *SD* standard deviation, *WBCT* whole-body computed tomography

^a^One patient did not undergo DXA because they were unable to fit in the scanner due to positioning of right arm

^b^CAJIS is the physician assessment of movement across 15 body regions (total score can range from 0 [normal] to 30 [functionally ankylosed across all regions])

^c^In seven patients with flare-ups during the preceding 12 months

There was a wide range of estimated radiation exposures for the low-dose WBCT scans (median: 13.7 millisieverts [mSv]; range 3.4–28.6 mSv). The estimated exposure for DXA was not calculated, since the manufacturer-reported dose was significantly lower than that for WBCT (≤ 0.03 mSv per scan).

### Evaluation of HO

**Fig. 3:**
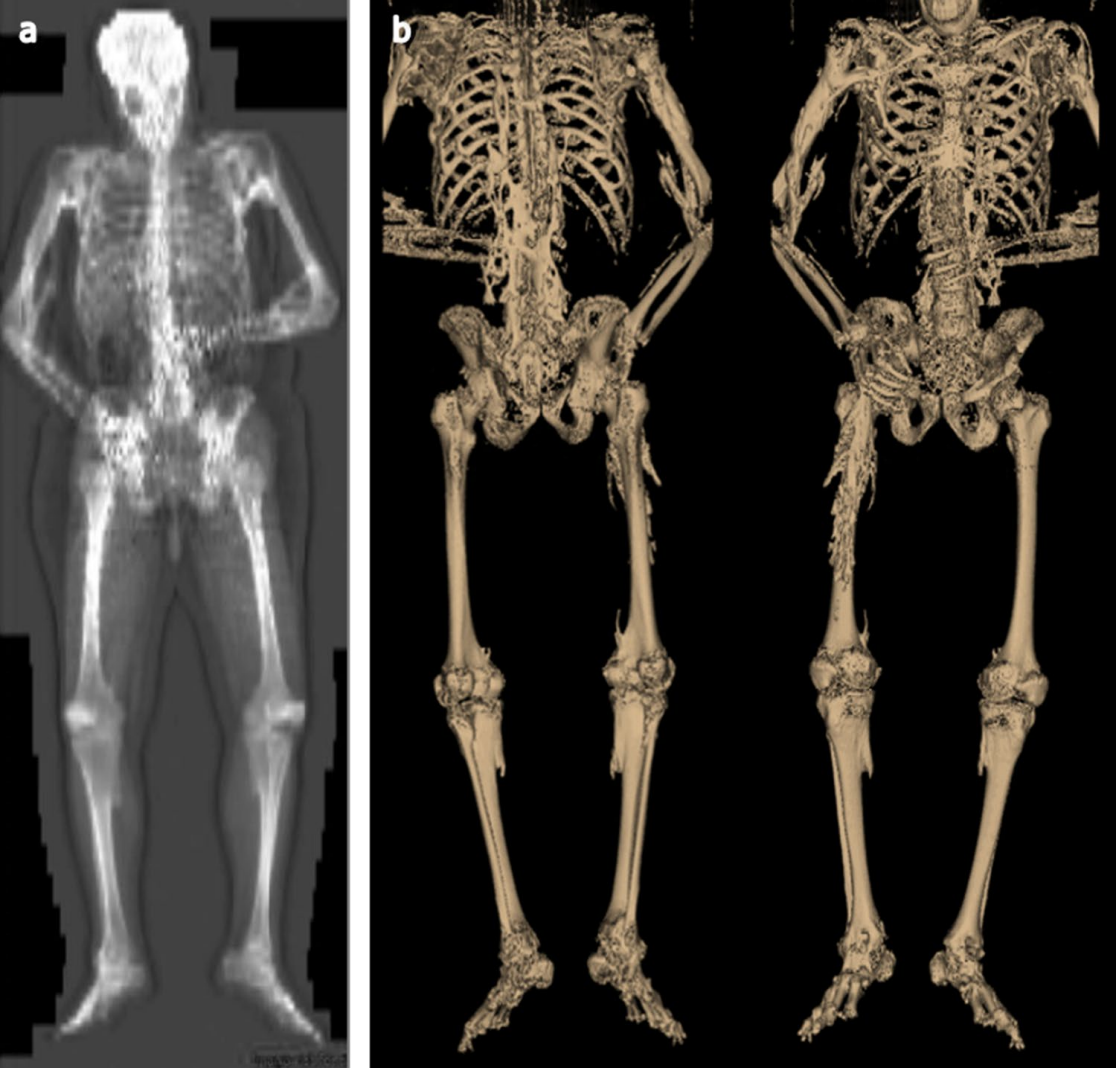
Representative **a** DXA and **b** 3D reconstructed WBCT scan (for demonstration only). Images were taken from the same 33-year-old male, with a Baseline CAJIS score of 18 at enrollment. The 3D reconstructed WBCT scan images were not used in the assessment or quantification of new HO in the analyses presented here, but are provided to demonstrate the amount of HO in a patient with FOP and to provide a similar view to the DXA images. *3D* three-dimensional, *CAJIS* Cumulative Analogue Joint Score, *DXA* dual energy X-ray absorptiometry, *FOP* fibrodysplasia ossificans progressiva, *WBCT* whole-body computed tomography

Representative low-dose WBCT and DXA scans are shown in Fig. 3. HO was primarily recorded in the axial regions (upper spine/chest and lower spine/abdomen), while absence of HO was most commonly observed in the extremities, consistent with known anatomic patterns of progression in FOP (Table 3) [23–25]. Most HO was mild but was more commonly moderate or severe in axial regions and hips than other body regions (Table 3).

**Table 3:** Comparison of extent and severity of HO with WBCT and DXA

Body region	HO evaluations, *n* (%)				
	HO non-evaluable	HO present	HO severity in evaluable regions with HO present^a^		
			Mild	Moderate	Severe
Head and neck^b^					
WBCT (*n* = 10)	2 (20)	6/8 (75)	6/6 (100)	0	0
DXA (*n* = 9)	4 (44)	1/5 (20)	1/1 (100)	0	0
Upper spine/chest					
WBCT (*n* = 10)	0	10/10 (100)	1/10 (10)	7/10 (70)	2/10 (20)
DXA (*n* = 9)	0	8/9 (89)	6/8 (75)	2/8 (25)	0
Lower spine/abdomen					
WBCT (*n* = 10)	0	8/10 (80)	4/8 (50)	4/8 (50)	0
DXA (*n* = 9)	0	6/9 (67)	5/6 (83)	1/6 (17)	0
Shoulders					
WBCT (*n* = 20)	0	12/20 (60)	8/12 (67)	4/12 (33)	0
DXA (*n* = 18)	1 (6)	9/17 (53)	9/9 (100)	0	0
Elbows					
WBCT (*n* = 20)	3 (15)	10/17 (59)^c^	7/10 (70)	1/10 (10)	0
DXA (*n* = 18)	4 (22)	6/14 (43)	4/6 (67)	2/6 (33)	0
Distal upper extremities					
WBCT (*n* = 20)	3 (15)	2/17 (12)	2/2 (100)	0	0
DXA (*n *= 18)	11 (61)	0	0	0	0
Hips					
WBCT (*n* = 20)	0	12/20 (60)	3/12 (25)	4/12 (33)	5/12 (42)
DXA (*n* = 18)	2 (11)	10/16 (63)	1/10 (10)	8/10 (80)	1/10 (10)
Knees					
WBCT (*n* = 20)	1 (5)	9/19 (47)	4/9 (44)	4/9 (44)	1/9 (11)
DXA (*n* = 18)	0	5/18 (28)	4/5 (80)	1/5 (20)	0
Distal lower extremities					
WBCT (*n* = 20)	3 (15)	7/17 (41)	6/7 (86)	1/7 (14)	0
DXA (*n* = 18)	0	2/18 (11)	2/2 (100)	0	0

*n* represents number of scans per region. Left and right limbs were imaged separately, but results presented here are combined

*DXA* dual energy X-ray absorptiometry, *HO* heterotopic ossification, *WBCT* whole-body computed tomography

^a^HO severity was only assessed in evaluable regions with HO present leading to variable n numbers

^b^For WBCT “head and neck” scans, the head was not included

^c^HO severity was non-evaluable in one WBCT scan for each of the left and right elbows

### Comparison of Imaging Modalities

**Fig. 4:**
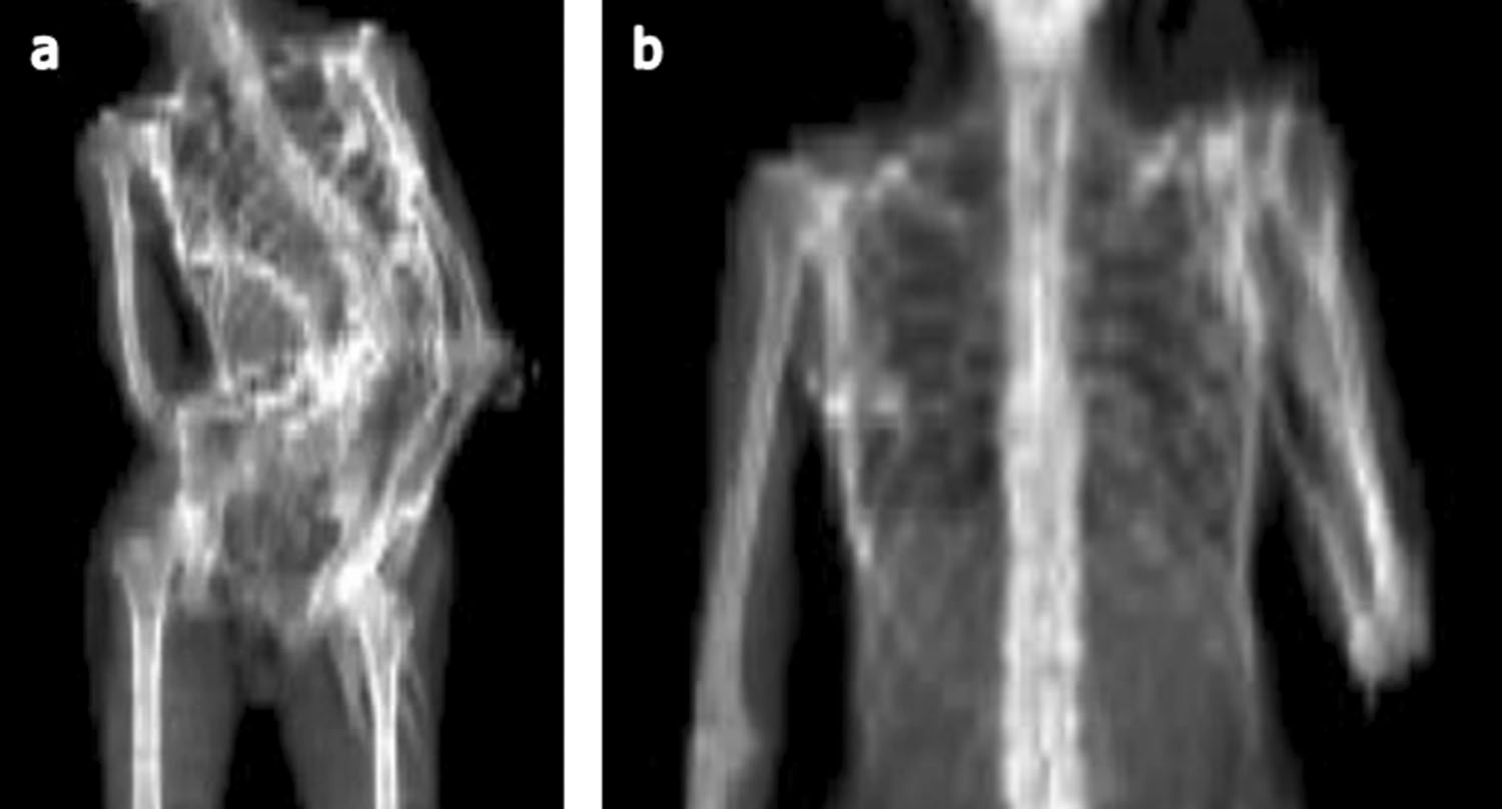
Overlapping anatomy precludes HO evaluation using **a** DXA or **b** CT scout scans. Image **a** was taken from an 18-year-old male, with a Baseline CAJIS score of 14 at enrollment. Image **b** was taken from a 34-year-old female, with a Baseline CAJIS score of 12 at enrollment. *CT* computed tomography; *DXA* dual energy X-ray absorptiometry, *HO* heterotopic ossification

During the assessment process, several regions could not be evaluated due to incomplete anatomical coverage in the field of view or overlapping anatomy in DXA scans (Fig. 4). In total, 60% of low-dose WBCT scans and 89% of DXA scans had ≥ 1 body region that was non-evaluable. Of the total 150 regions (15 from 10 scans) to be assessed in WBCT, 12 (8%) were non-evaluable: 2 neck, 3 elbow, 3 hand/wrist, 1 knee, and 3 ankle/foot. The mean number of non-evaluable regions per WBCT scan was 1.2 (SD: 1.5). Of the 135 regions (15 regions in 9 scans) assessed in DXA, 22 (16%) were non-evaluable: 4 head/neck, 1 shoulder, 4 elbow, 11 hand/wrist, and 2 hip. The mean number of non-evaluable regions per DXA scan was 2.4 (SD: 1.4). Thus, a higher proportion of DXA scans and regions were considered insufficient for evaluation of HO presence compared with WBCT. This difference was primarily due to the high number DXA of scans with non-evaluable distal upper extremities as a result of contractures of the upper limbs (Table 3); in other regions, detection of HO was similar between DXA and WBCT.

The evaluation of HO presence and severity varied considerably between the two imaging modalities (Table 3), with WBCT indicating presence of HO in 76/138 (55%) of evaluable body regions compared with 47/113 (42%) for DXA. This was particularly evident, for example, in scans of the head/neck, for which WBCT (neck only) indicated presence of HO in the majority of patients (*n* = 6/8 [2 non-evaluable]), while DXA did so in only 1 out of 5 patients (4 non-evaluable), despite the fact that the proportion of scans with this region being evaluable was comparable between the two modalities. This was most likely due to the three-dimensional aspect of WBCT which allows for the detection of small amounts of HO; the two-dimensional aspect of DXA may not detect small amounts of HO overlying normotopic bone. Such discrepancies were less apparent across other body regions (Table 3). Similarly, a higher proportion of HO (where present) was assessed by WBCT as moderate or severe in the upper spine/chest (*n* = 9/10) and lower spine/abdomen (*n* = 4/8) than with DXA (upper spine/chest: *n* = 2/8; lower spine/abdomen: *n* = 1/6) (Table 3). Additionally, only WBCT allowed for three-dimensional evaluation, and therefore quantitative assessments of volume (Fig. 3b). Median HO volume of the 10 patients was 426,209 mm^3^ (range: 48,844–1,515,484 mm^3^).

## Discussion

When evaluating disease progression in patients with FOP, it is preferable to visualize the location and severity of HO and quantify changes in whole-body HO volume over time. Our findings indicate that low-dose WBCT (excluding the head, to minimize radiation exposure to brain and surrounding structures) is most appropriate for documenting the presence, location, and total body volume of HO. The International Clinical Council on FOP has also endorsed volumetric assessment of ossification using low-dose CT as a common endpoint for clinical trials [26].

Both DXA and low-dose WBCT were selected for evaluation in this study based on radiation exposures, instrument availability, and practical constraints. Specifically, whole-body DXA imaging was evaluated based on the simplicity of acquisition, the wide availability of instruments, and the relatively low radiation exposure (0.3 mSv). In addition, whole-body DXA is routinely performed in pediatric populations, so normative age-related data are available to assist with the detection of exogenous total bone, including mineralized tissues outside of the endogenous skeleton [27, 28]. Finally, the open nature of a DXA instrument was potentially more conducive to accommodating the sometimes awkward limb positioning that results from joint ankylosis in patients with FOP.

In this pilot phase of the NHS, DXA scans provided adequate visibility of HO in body regions that did not overlap the normal skeleton in the coronal plane. However, patients with FOP experience ankyloses of joints in a cranial-to-caudal and axial-to-appendicular pattern, often locking them in positions where multiple body regions may overlap [29]. Where HO overlapped the normal skeleton, it was not possible to distinguish normal from heterotopic bone, precluding accurate HO identification and monitoring. As a result, there was a higher percentage of patients with non-evaluable regions with DXA compared with WBCT imaging.

Where regions were evaluable, results indicated that DXA is less sensitive than WBCT in the evaluation of the presence and severity of HO, especially in the neck and axial regions. It was also not possible to assess HO volume using DXA due to its two-dimensionality. Thus, while DXA scanning has the benefit of simplicity, relatively low radiation exposure and quick acquisition time, the two-dimensional nature had a substantial negative impact on the HO evaluation, and in particular, HO change over time. Nevertheless, DXA may be the preferred imaging modality to avoid radiation exposure in children; since HO progresses with age, children are also less likely to have the contractures of the limbs that make distinguishing heterotopic from normotopic bone more difficult.

Overall, WBCT was determined to be the modality of choice due to its three-dimensionality, and the reduced number of regions with non-evaluable HO compared with DXA scans. WBCT is not commonly used in clinical imaging; axial PET-CT is used as part of standard care in oncology and has been used to monitor HO in FOP [20], but it typically does not include the appendicular skeleton. To evaluate total body burden of HO in this NHS, WBCT scans were required to include the torso and upper and lower extremities. It was equally important to keep the radiation exposure as low as possible, without compromising image quality. A few prior oncologic studies have demonstrated the utility of low-dose WBCT: Alessio et al*.* (2009) reported reductions in radiation dose of 8.0–13.5 mSv for whole-body PET/CT scans in pediatric patients (20–50% less than the standard fixed CT technique, using 120 mAs and 120 kVp) [30]; Horger et al*.* (2005) examined the use of low-dose WBCT for the diagnosis of lytic bone changes and for the assessment of fracture risk in patients with multiple myeloma, and described the acquisition of diagnostic scans at low doses (4.1 mSv) by significantly reducing the energy level settings (40 mAs) [31].

To limit radiation exposure in this NHS, the use of initial scout scans without subsequent axial scans was considered, but this has similar limitations to DXA scans for HO evaluation. Instead, the NHS Part A protocol and imaging guidelines provided to study sites required that WBCT scans be of low radiation dose. This was achieved by a central imaging laboratory providing acquisition parameters to each study site, and the subsequent estimation of radiation exposure and review of image quality by a medical physicist after the acquisition of each scan. Feedback was subsequently provided to each study site, as necessary, to recommend the modification of the acquisition parameters to reduce radiation exposure, while maintaining image quality. Despite this, a wide range of total exposures (3.4–28.6 mSv) was initially identified. Therefore, further guidelines were provided for Part B of the NHS: following the scout acquisition, the technologists were instructed to check the predicted CTDI_v_ for the axial scan and to further adjust settings so that this was < 5 milligrays (mGy); emphasis was placed on proper patient positioning at the iso-center (both horizontal and vertical axes), in order to avoid noisy images and a corresponding increase in radiation dose due to automated exposure control. Careful attention to CT scan dose is an important factor when considering the use of WBCT given that even the optimized lower dose of approximately 4 mSv is similar to the estimated average annual exposure in the USA to naturally occurring radioactive materials and cosmic radiation from outer space [32].

The relative cost of WBCT is greater than that of whole-body DXA (the former being more than twice as expensive in the USA). This can make imaging with WBCT prohibitively expensive for use in clinical trials. However, the availability of CT and DXA scanners is comparable, meaning that the access to the two modalities is not a limiting factor. Patient burden during scans was also considered to be broadly comparable between WBCT and DXA, since both require the patient be positioned supine on a padded table, to lie still for a few minutes while the scan is acquired, and allow for normal breathing.

Patients with FOP often have anatomic deformities that limit their ability to lay flat when supine or have body parts extending outside the field of view. In addition, patients with FOP are at high risk of injury from falls, bumps, or moving parts of the imaging equipment; such trauma can lead to consequences such as a FOP flare-up and subsequent HO. CT scanners typically have a field of view of 50 cm diameter, which sufficiently accommodates most human bodies. DXA scanners have a maximum width of 58–67 cm on average and can fit human bodies with a body mass index < 30 kg/m^2^. In the population of individuals with FOP enrolled in Part A of the NHS, several patients had ankylosis deformities in the extremities (knees and elbows) that restricted the legs and arms from being positioned straight and flat on the scan table. In some cases, this resulted in extremities extending outside the scan field of view for both WBCT and DXA, leading to non-evaluable sub-regions. This limitation occurred in both imaging modalities, and could require repositioning or split scanning (separate upper and lower body scans) for proper imaging.

A limitation of the study overall was the inability to evaluate more than two imaging modalities, although the two that were evaluated were selected based on radiation exposures, availability and practical constraints. In particular, ^18^F-NaF PET-CT was not considered for evaluation in this study, as it was not widely available at the time this study was designed. However, the potential value of ^18^F-NaF PET-CT imaging modality has been described more recently [20].

## Conclusion

In this study, low-dose WBCT (excluding the head) was determined to be the preferred method for assessing total body burden of HO in clinical studies of patients with FOP. A major benefit of WBCT was the elimination of overlap in the central or axial regions where the largest HO burdens are found. This information was carried forward to Part B of the NHS, which enrolled more than 100 patients with FOP to evaluate disease progression over three years [33]. The data gathered from this NHS will help to identify clinically meaningful endpoints.

## Data Availability

Where patient data can be anonymized, Ipsen will share all individual participant data that underlie the results reported in this article with qualified researchers who provide a valid research question. Study documents, such as the study protocol and clinical study report, are not always available. Proposals should be submitted to DataSharing@Ipsen.com and will be assessed by a scientific review board. Data are available beginning 6 months and ending 5 years after publication; after this time, only raw data may be available.
